# Nexplanon failure in a woman with HIV infection in rural Ghana: A case report

**DOI:** 10.1002/ccr3.3156

**Published:** 2020-07-16

**Authors:** Maxwell Tii Kumbeni, Paschal Awingura Apanga, Emmanuel Awine Ayamga

**Affiliations:** ^1^ Ghana Health Service Nabdam District Health Directorate Nangodi Ghana; ^2^ Ghana Health Service Talensi District Hospital Tongo Ghana

**Keywords:** efavirenz, Ghana, Nexplanon, unplanned pregnancy

## Abstract

Although Nexplanon is one of the most effective and most utilized long‐acting reversible contraceptives in Ghana. We report a rare event of Nexplanon failure in a woman with human immunodeficiency virus (HIV) infection in rural Ghana.

## Graphical abstract

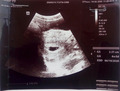

## 1 INTRODUCTION

Family planning is considered globally as an important intervention toward attaining the sustainable development goals (SDG) target 3.7, which is focused on preventing unplanned pregnancy and reducing teenage childbirth through universal access to sexual and reproductive health services.^1^ The United Nations (UN) has lauded the rise on contraceptive use, particularly the use of long‐acting or permanent contraceptive methods among married couples.^2^ The UN reported that more than one‐third of married women globally are on contraceptive methods such as subdermal implants, intrauterine devices (IUD), and sterilization.^2^ The use of long‐acting reversible contraceptives (LARCs) has led to significant lower rates of unplanned pregnancies as compared to short‐acting reversible contraceptives (SARCs).^3^, ^4^ LARCs also have higher satisfaction and adherence rates compared to SARCs.^5^ The use of LARCs have also been effective in preventing unplanned pregnancies during postpartum or postabortion.^6^, ^7^

The use of subdermal implants to prevent unplanned pregnancies has increasingly become prevalent globally including Ghana.^2^ Nexplanon is a long‐acting reversible subdermal contraceptive implant which has been proven as a highly efficacious contraceptive device.^8^, ^9^ It is cost effective, convenient to use, and highly efficacious compared to other contraceptive methods.^10^, ^11^ Return to fertility is also quick with Nexplanon and can safely be used by breastfeeding mothers.^10^, ^12^ It can also be used by women who are not tolerant to estrogen.^12^ Despite its benefits, clients on Nexplanon have reported adverse effects, discontinued its use, and have reported contraceptive failure.^9^, ^13^, ^14^ Most of these “failures” are associated with faulty insertion technique, client already pregnant, insertion at the wrong time during the menstrual cycle, expulsion of the implant, and drug interactions.^15^, ^16^

We present a rare case of Nexplanon failure in a woman with HIV infection in rural Ghana. There are limited studies on the failure of subdermal contraceptive implants such Nexplanon in clients with HIV infection.^13^ To the best of our knowledge, Nexplanon failure has also not been reported in a woman with HIV infection in rural Ghana.

## 2 CASE PRESENTATION

The client is a 37‐year‐old widow with weigh of 68kg and was diagnosed of HIV infection in 2014 and has been on the following antiretrovirals: Tenofovir (300 mg); Lamivudine (300 mg); and Efavirenz (600 mg). Diagnosis of HIV infection was made using first response rapid diagnostic HIV test kits and confirmed with OraQuick test. The diagnosis of HIV infection and regimen of treatment in this woman was based on local protocols. Her parity is six with four children alive with two infant deaths. All six gestations were carried to term, uneventful with spontaneous vaginal deliveries. She has a regular menstrual cycle of 28 days. She has no history of other chronic diseases such as diabetes, hypertension, and epilepsy. She has no history of contraceptive failure including Nexplanon. She is not on any other medications except the antiretrovirals. She also has no history of smoking or alcohol consumption.

The client presented to the family planning clinic of a health facility in rural Ghana on the 6th of December 2017 and requested for Nexplanon (68 mg Etonogestrel) after informed counseling on all available contraceptive methods. Her last menstrual period was on 30th November 2017, and a urine pregnancy test was also done to rule out pregnancy. The client was eligible for Nexplanon based on the WHO Medical Eligibility Criteria for contraceptive use.^17^ An experienced trained community health nurse on LARCs inserted the Nexplanon into her upper nondominant arm (left arm) under aseptic conditions on the same day. The client returned for review in a month's later without any complains. The expiry date of the Nexplanon inserted was December 2020.

**Figure 1:**
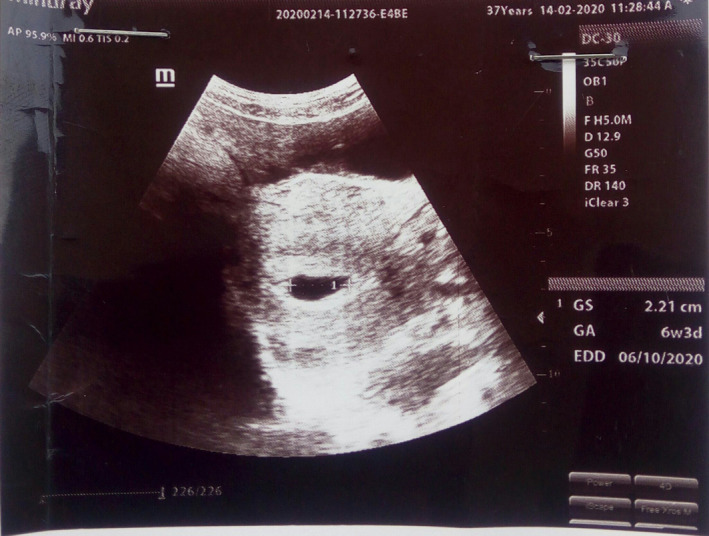
Obstetric ultrasound scan taken on 14th February 2020. Gestational sac (GS) was 2.12 cm, and gestational age (GA) was 6 weeks and 3 days according to the scan. Expected date of delivery was 6th October 2020

On the 13th of February 2020, the client reported to the health facility with the complains of amenorrhea for six weeks, feeling of breast heaviness and dizziness. Client tested positive for pregnancy at the health facility. An obstetric ultrasound scan confirmed a single viable intrauterine gestation at six weeks and three days with no visible fetal pole yet (Figure 1). This was an unplanned pregnancy, and client requested for termination of pregnancy after counseling. On examination of the left upper arm, the Nexplanon rod was well positioned and rod was removed. Client's pregnancy was terminated via manual vacuum aspiration after obtaining informed consent. After the termination of the pregnancy, client against medical advice refused to be put on any form of contraception. However, the client returned to the family planning clinic of the health facility on the 22nd February 2020. She chose Jadelle, and this was inserted for her after informed counseling was done.

## 3 DISCUSSION

Drug interactions with LARCs can reduce the efficacy of contraceptives.^18^ The cytochrome P450 (CYP) enzyme system in the liver plays a crucial role in drug metabolism, and drugs that induce these enzymes are capable of causing increased elimination of hormonal contraceptives, leading to reduced efficacy and exposing the client to a high risk of unplanned pregnancies.^19^ Some common enzyme inducers include antiepileptic drugs (phenytoin, phenobarbital, oxcarbazepine, topiramate, and carbamazepine) ^20^; antitubercular drugs (rifampicin and isoniazid); and non‐nucleoside reverse transcriptase inhibitors (efavirenz and nevirapine).^21^, ^22^ These drugs induce the CYP450 system which enhances the metabolism of estrogen and progestin and thereby reducing the serum concentration of progestin.^20^ Such drugs have led to sporadic cases of subdermal implant failure which have resulted into intrauterine or ectopic pregnancies.^3^, ^13^, ^14^

This client was treated with an Efavirenz (EFV)‐based antiretroviral while on Nexplanon. Etonogestrel is metabolized by cytochrome P450 3A4 (CYP 3A4), and EFV enhances the systemic clearance of coadministered drugs that are cytochrome CYP 3A4 substrates.^23^ Thus, EFV might have led to reduced serum levels of etonogestrel in our client leading to her unplanned pregnancy. This is a possible as recent studies have suggested that women on Nexplanon contraception and EFV‐based antiretrovirals have a lower serum concentration of etonogestrel plasma concentration as compared with women who are not on antiretrovirals.^24^, ^25^, ^26^ EFV‐based antiretrovirals are known to reduce the etonogestrel serum concentration threshold for ovulation suppression which reported as 90 pg/mL.^24^, ^26^ This increases the risk of ovulation among clients that combine the use of EFV‐based antiretrovirals and Nexplanon.

Studies have reported Nexplanon failure in patients on EFV.^27^, ^28^ The failure of implants due to its interaction with EFV has the potential to result in many unwanted pregnancies.^27^ It can also undermine the confidence women have on implants. To avoid unwanted pregnancies, it has been suggested by Shelton to replace EFV with another ARV that does not significantly reduce progestin blood levels.^27^

Although Nevirapine and EFV are inducers of CYP 3A4, these drugs are part of the recommended first‐line regimen for treatment of people living with HIV including women of reproductive age according to the WHO.^29^ Women of reproductive age living with HIV and on EFV‐based antiretrovirals are also eligible to receive Nexplanon.^17^ Though our client's treatment of HIV infection and use of Nexplanon were consistent with the WHO guidelines on antiretrovirals and contraceptive use, clients on EFV‐based antiretrovirals may not be suitable candidates for Nexplanon use due to the interactions between EFV/ Nevirapine and etonogestrel.

## 4 CONCLUSION

This case report emphasizes the possibility of unplanned pregnancy with the concurrent use of Nexplanon and EFV‐based antiretrovirals although the WHO medical eligibility criteria permit the use of Nexplanon in women who are on EFV‐based antiretrovirals. However, further studies are needed to establish whether a causal relationship exists between EFV‐based antiretrovirals and Nexplanon failure.

## CONFLICT OF INTEREST

None declared.

## AUTHOR CONTRIBUTIONS

MTK and EAA: involved in care of the patient. MTK and PAA: prepared the manuscript. EAA: edited the manuscript. All authors: read and approved the final manuscript.

## CONSENT FOR PUBLICATION

Written informed consent was obtained from the patient for publication of this case report and its accompanying image. A copy of the written consent is available for review by the Editor‐in‐Chief of this journal.
